# Bouveret Syndrome: A Rare Case of Instance and Treatment in a Younger Patient

**DOI:** 10.1155/2020/1837387

**Published:** 2020-03-16

**Authors:** Kevin J. Yang, C. K. Chang

**Affiliations:** ^1^University of California, Berkeley, CA, USA; ^2^Kaiser Permanente, Oakland, CA, USA

## Abstract

Bouveret syndrome, a specific form of gallstone ileus, is the obstruction of the gastric outlet by a gallstone, which can enter the duodenum through a fistula. While the average age of individuals with Bouveret syndrome is 74 years, our patient was 42 years of age at the time of operation, significantly younger than the average patient afflicted with this condition. In the treatment of our patient's condition, the operation conducted entailed a partial duodenectomy, gastrojejunostomy, cholecystectomy, common bile duct exploration, extraction of bile duct stones, and insertion of a t-tube in the bile duct. The patient was found to be in healthy condition upon check-up six months after the operation. The outcome of our case suggests that younger Bouveret patients can safely undergo multiple surgical procedures in the treatment of Bouveret syndrome. Our case also suggests that a cholecystectomy and the removal of the obstructing gallstone can both be carried out within one operation, although coupling these two procedures in one operation might be riskier for patients within the normal age range of Bouveret syndrome. We also suggest that fistula repair be carried out for younger Bouveret patients in particular and that the patient be subjected to a CT scan in the diagnosis of Bouveret syndrome when this condition is suspected.

## 1. Case Presentation

Bouveret syndrome, a specific form of gallstone ileus, is the obstruction of the gastric outlet by a gallstone, which can enter the duodenum through a fistula. Bouveret syndrome most often occurs in individuals of advanced age; 74 years old is the average age of Bouveret syndrome patients [1]. This report summarizes and discusses the treatment of Bouveret syndrome in a patient who is 42 years of age at the time of operation, well below the age of the average Bouveret syndrome patient.

**Figure 1:**
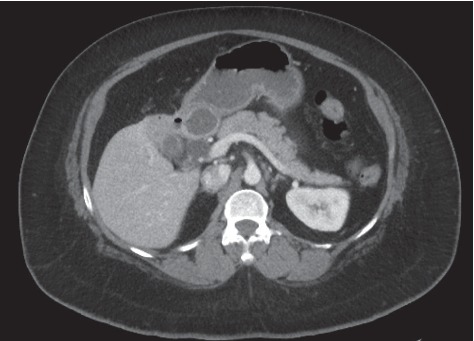
CT scan showing a large stone in the duodenum and a cholecystoduodenal fistula.

**Figure 2:**
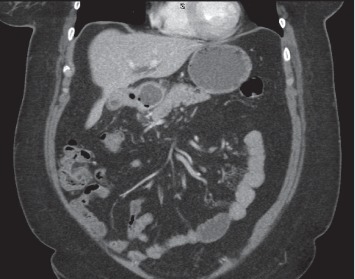
Addition CT scan showing a large stone in the duodenum and a cholecystoduodenal fistula.

The patient is a 42-year-old female who presented with a gastric outlet obstruction. A CT scan showed a 2.5 × 5 cm stone in the duodenal bulb with a cholecystoduodenal fistula (Figures 1 and 2). An upper endoscopy was performed which showed a large gallstone in the duodenal bulb, causing complete obstruction of the duodenum. The gallstone could not be removed endoscopically. Surgical exploration identified a large stone in the duodenum which could not be mobilized proximally or distally. A cholecystectomy was performed, confirming the presence of the large cholecystoduodenal fistula identified in the CT scan. The fistula was 3 cm in diameter. A partial duodenectomy, a gastrojejunostomy, a cholecystectomy, and a common bile duct exploration with extraction of multiple common bile ducts stones were performed. A t-tube was placed into the bile duct after bile duct exploration. A follow-up t-tube cholangiogram was performed four weeks after the operation, revealing no strictures or additional stones. The t-tube was removed at six weeks after the operation. The patient was found to be in healthy condition upon check-up six months after the operation.

## 2. Discussion

At 42 years of age, our patient is well below the age of 74, the average age of individuals who are treated for Bouveret syndrome [1]. Our patient affords the unique opportunity to examine the treatment of a younger individual afflicted by this condition.

Existing literature warns against carrying out anything beyond an enterolithotomy in the treatment of Bouveret syndrome if endoscopic methods fail, as the advanced age of most Bouveret patients and the conditions associated with advanced age most often render these patients poor surgical candidates [2]. Such a conclusion suggests that younger Bouveret patients should be able to safely undergo multiple procedures in one surgery, but this is not self-evident and requires more support. Our patient underwent five major procedures (partial duodenectomy, gastrojejunostomy, cholecystectomy, bile duct exploration, and the extraction of stones in the common bile duct) within one surgery and is in good health as of six months after the operation, with no complications between the operation and the time of writing. As such, the outcome of our case supports the statement that younger Bouveret patients can safely undergo multiple surgical procedures in the treatment of Bouveret syndrome, a course of action which can reduce the risk of gallstone recurrence [2].

Our case corroborates several existing trends in the literature concerning the surgical treatment of Bouveret syndrome in the context of a younger patient. The current literature suggests that one of the most effective, if not the most effective, imaging technique to diagnose Bouveret syndrome is the CT scan [1, 3–5]. In the diagnosis of our patient, the CT scan successfully revealed both a large stone within the duodenal bulb and its associated cholecystoduodenal fistula. As such, our case, alongside the aforementioned existing literature, demonstrates that the CT scan is an efficacious means by which to diagnose Bouveret syndrome. Furthermore, reviews of the literature have stated that endoscopy, while being the least invasive (and therefore most desirable) method to remove a Bouveret syndrome gallstone, is rarely successful [6, 7]. We, too, were unable to remove the gallstone endoscopically, necessitating full surgical intervention. Thus, while it remains that endoscopic methods may be useful as a diagnostic tool, surgeons presented with the task of treating Bouveret syndrome should be prepared to remove the gallstone surgically.

Our case may have implications with regards to the ongoing debate over whether or not to carry out fistula repair when addressing Bouveret syndrome via surgical intervention [3, 5–7]. Evidence against fistula repair includes the fact that Bouveret syndrome patients who did not undergo fistula repair have lower mortality rates and the theory that such fistulas will close spontaneously [5, 7]. Evidence in favor of fistula repair includes reduction in the chance of a number of postoperative complications, such as the recurrence of gallstone ileus and cancer risk [3]. In our particular case, fistula repair was carried out, with positive results evidenced by the healthy condition of the patient six months after the operation. This course of action was taken considering the age of the patient; being much younger than most patients afflicted with Bouveret syndrome, our patient would most likely have a much lower chance of mortality associated with fistula repair than that expressed in the literature, as this mortality rate is based on a patient population with a much higher mean age. Moreover, our patient's young age means that she would be at a greater risk of developing those problems associated with a persisting fistula, as this fistula would persist in our patient for the entire duration of the patient's life before death if spontaneous closure of the fistula does not occur. These factors lead us to recommend that fistula repair be carried out in the surgical treatment of Bouveret syndrome in younger patients.

Furthermore, our case may have implications with regards to an ongoing debate over the treatment of the broader condition of gallstone ileus. The present controversy in the literature is over whether to remove the obstructing gallstone and perform a cholecystectomy in one surgery or in two separate surgeries [8]. In our case, these two procedures were carried out within one surgery with positive results, lending support to a “one surgery” approach. However, additional studies of cases are necessary to lend such a statement reasonable credibility, especially considering the fact that our patient is well below the age of the average gallstone ileus patient [9].

Our recommendations fall in line with the successful treatment of Bouveret syndrome in patients below the age of 50. Those cases documenting a cholecystectomy performed this procedure within the same operation as the removal of the gallstone, with successful results [10–13]. Furthermore, all cases of patients below the age of 50 carried out a fistula repair, again with successful results [10–14]. As such, our case, alongside the existing literature regarding treatment in younger patients, shows that younger patients indeed can accept the greater surgical pressures associated with these approaches.

Our case offers the rare opportunity to study the successful treatment and outcome of a Bouveret syndrome patient whose age is far below that of the average patient afflicted by this condition. Existing literature finds that the old age of most Bouveret patients makes them unfit to undergo numerous surgical procedures in the treatment of Bouveret syndrome; however, the inverse of this conclusion, that a younger Bouveret patient is fit for a greater number of procedures within one surgery, lacks support in the existing body of literature. Our case lends support to the statement that a young patient can undergo a more elaborate set of procedures in the treatment of this condition. With regard to two present controversies in the literature, we recommend performing cholecystectomy in the same surgery as the gallstone removal when cholecystectomy is deemed necessary and to carry out fistula repair when treating young Bouveret patients. Lastly, we recommend employing a CT scan to diagnose Bouveret syndrome when this condition is suspected.
